# Laparoscopic management of appendicular mass

**DOI:** 10.4103/0972-9941.78345

**Published:** 2011

**Authors:** Vishwanath V Shindholimath, K Thinakaran, T Narayana Rao, Yenni Veerabhadrappa Veerappa

**Affiliations:** Department of Surgery, PES Institute of Medical Sciences and Research, Kuppam, 517 425, District Chittoor, Andhra Pradesh, India; 1 Department of Pathology, PES Institute of Medical Sciences and Research, Kuppam, 517 425, District Chittoor, Andhra Pradesh, India

**Keywords:** Appendicular mass, Laparoscopy

## Abstract

**BACKGROUND:**

Laparoscopic appendectomy is becoming the preferred technique for treating acute appendicitis. However, its role in the treatment of complicated appendicitis is controversial. This study was undertaken to assess the feasibility of laparoscopic appendectomy for appendicular mass.

**MATERIALS AND METHODS:**

A retrospective review was performed of all the patients who were treated laparoscopically for appendicular mass from March 2007 to October 2009. Setting: Tertiary care hospital.

**RESULTS:**

A total of 120 patients were treated for appendicitis. A retrospective review of the patients’ records demonstrated that 19 patients (15.8%) had appendicular mass at the time of admission. The average operative time was 95 minutes (range 45-140 minutes). Pathological evidence of appendicitis was present in all the patients. The average length of hospital stay was six days (rang 6-9 days). Three patients (15.7%) had post- operative complications. Two patients developed wound infections and one patient was re-admitted with pain and a lump below the umbilical port.

**CONCLUSION:**

The findings suggest that laparoscopic appendectomy is feasible in patients with appendicular mass. The authors propose a prospective, randomized trial to verify this finding.

## INTRODUCTION

Acute appendicitis remains the most common intra-abdominal surgical pathology requiring surgical intervention. The cases of patients presenting late in the course of acute appendicitis are complicated by the development of an inflammatory mass in the right iliac fossa. The treatment of appendicular mass is controversial. It is changing from the traditional approach of initial conservative treatment followed by interval appendectomy to immediate appendectomy.[1,2]

Early surgical intervention has been known to is known to be an effective alternative to conservative therapy for a long time, as it considerably reduces the total hospital stay and obviates the need for a second admission.[3] Moreover, in 10-20% of the cases, conservative management fails and the patients need an emergency operation due to peritonitis, which is comparatively more difficult and carries more morbidity and mortality.[4,5] In addition, the patient may suffer a recurrence of appendicitis after being discharged from the hospital.[6,7] In rural areas, a large number of patients refuse an operation once their acute problem is solved and this seems to be a major disadvantage of the initial conservative approach. Another disadvantage of the conservative management is the chance of misdiagnosis like intussusceptions and carcinoma caecum may be treated conservatively by mistake adding considerable morbidity.[8] The early operation on the other hand has an edge of being curative in the index admission and ensures early return to-work and higher compliance. Controversy is not only confined to management, but is also about the technique, laparoscopy versus open.

For decades, open all forms appendectomy has been the standard treatment for all forms of appendicitis.[9,10] Generous right iliac fossa incisions were taken as the appendectomy could be difficult due to adhesion, omental wrapping, and abscess formation within the mass. Since its description in the early 1980s, laparoscopic appendectomy has become an acceptable approach for simple appendicitis.[11–13] However, its role in the treatment of complicated appendicitis is controversial. We conducted this study to assess the safety and efficacy of laparoscopic appendectomy in patients with complicated appendicitis in our institution.

## MATERIAL AND METHODS

All the patients who underwent laparoscopic appendicectomy between March 2006 and October 2009, at our institute, were retrospectively reviewed. A total of 120 patients were treated for appendicitis. A retrospective review of the patients’ records, while they were in the hospital, demonstrated that 19 patients (5.6%) had an appendicular mass when they were admitted to the hospital.

All the operations were performed using the three-trocar technique. Endoscopic pre-tied loops were used for ligation of the base of the appendix. Injection-Cefotaxime 50 mg/kg six hourly, Amikacin 2.5 mg/kg eight hourly and metronizadole 7.5 mg/kg eight hourly were given intravenously, for five days. All areas of the intra-abdominal collection were aspirated and the peritoneal cavity was rinsed with normal saline. An abdominal drain was kept in 13 patients. Ten patients were discharged on sixth post-operative day, with oral antibiotics (cefixime + metronidazole) for another five days; and nine patients stayed for more than seven days. Data pertaining to sex, age, duration of symptoms, operative time, operative findings, complications, length of hospital stay, and pathological results were reviewed.

## RESULTS

During the study period 19 laparoscopic appendectomies were performed for appendicular mass. There were thirteen male and six female patients in this series; the male-to-female ratio was 2:1. The patients ranged in age from 12 to 45 years. The duration of treatment elsewhere, before the admission, ranged from four day to ten days. All but 15 patients were febrile when they were admitted to the hospital, and most patients (66%) had leukocytosis of greater than 11,000 per mm^3^. All 19 patients underwent laparoscopic appendectomies within 24 hours of admission.

**Figure 1:**
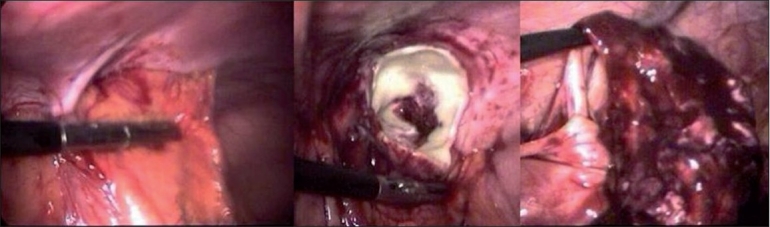
Appendicular abscess cavity and appendix being separated from the mass

The average operative time was 95 minutes (range 45-140 minutes). The post-operative analgesia requirement was minimal. The average length of hospital stay was six days (rang 6 - 9 days). There were no intra-operative complications. The findings at surgery are listed in Table 1. In 12 patients there was difficulty in localisation of the appendix [Table 2]. There was one conversion that was excluded from further analysis. The reason for conversion was appendicular perforation with unhealthy appendicular base and cecal gangrene, for which we did hemicolectomy. Three patients (15.7%) had post-operative complications. Two patients developed minor wound infections and one patient was re-admitted with pain and a lump below the umbilical port. An ultrasound reveled omental mass adherent to the peritoneum near the umbilicus. There was no collection. In this case unhealthy omentum, which had formed an abscess, was not removed [Figure 1]. The patient was treated conservatively with antibiotics and the mass disappeared after 10 days. Examination findings were normal in all up to 6 months after discharge. The patients did not report any problems during follow-up.

**Table 1:** Per-operative findings

Operative findings	Nos.
Appendicular abscess	6
Perforated appendix	7
Loculated pus collection	1
Gangrenous appendix	5

**Table 2:** Intra-operative Problems

Operative problems	Nos.
Difficulty in localisation of appendix	12
Difficulty in adhenolysis	6
Bleeding	1

One patient who had a post tubectomy intra-abdominal stitch abscess in the right iliac fossa, which was treated with 10 days of antibiotics, was excluded from the study. During laparoscopy we found that the appendix was adherent to the mass but was not involved in it. There were dense adhesions among the peritoneum, cecum, and right fallopian tube; when gradually separated we found a small abscess cavity containing two threads and pus, which was removed and appendectomy was performed. The patient did well in the post-operative period.

## DISCUSSION

Many controversies exist regarding the optimal management of patients with appendicular mass. These include conservative treatment versus surgery, duration of antibiotic therapy, drain usage and skin closure. Of late, the question of performing laparoscopic appendectomy for appendicular mass has been added to the list of controversies.

**Figure 2:**
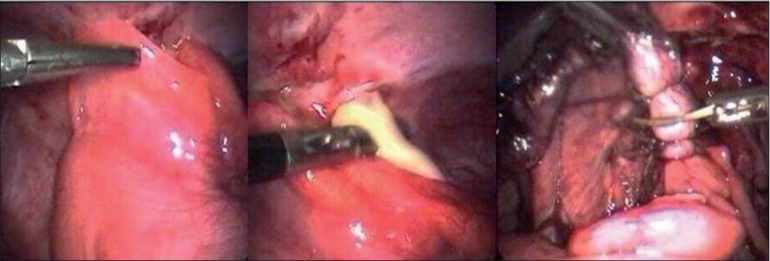
Appendicular abscess and dissected appendix being cut

It is not always possible to distinguish between an appendiceal mass and an appendiceal abscess before the operation [Figure 2]. And non operative management is not always successful.[14–16] In Thomas’ series, six of 37 patients thought to have a phlegmon were subsequently found to have abscesses.[14] Another disadvantage of the conservative management is the chance of mis-diagnosis as reported by Garg *et al*., ^8^ who claim that conditions like intussusception and carcinoma caecum may be treated conservatively by mistake adding considerable morbidity. Similarly, in our study also, the post tubectomy stitch abscess presenting as a right iliac fossa mass, with fever, was misdiagnosed as appendicular mass and managed with antibiotics for more than two weeks.

**Figure 3:**
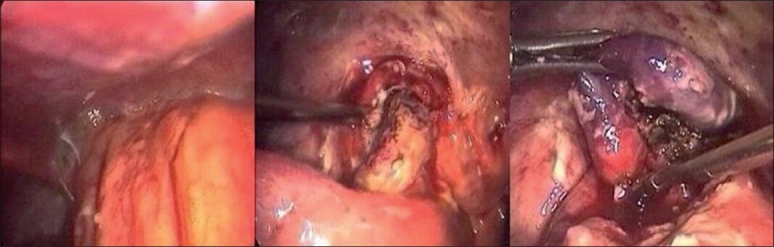
Appendicular mass and gangrenous appendix

Early operation on the other hand has an edge of being curative in the index admission and ensures early return- to-work and higher compliance. The earlier belief that surgery is difficult in a state where the inflamed appendix is buried deep in the mass and the bowel loops are friable is no more a valid argument at present, due to an improvement in anaesthesia, electro surgical unit and antibiotics. The operative problems such as localisation of the appendix, adhenolysis and bleeding can be tackled with a magnified view of the laparoscope [Figure 3].

Jordan *et al*., in 1979, performed 42 open appendicectomies in palpable masses and recommended early surgery in patients with appendicitis with palpable mass.[3]

Horwitz *et al*.,[17] and others have suggested avoiding the laparoscopic approach in complicated appendicitis because of the increased risk of post-operative, intra-abdominal abscesses.[18,19] None of our patients developed such complications. Several factors might have contributed to this result. All procedures were performed by experienced laparoscopic surgeons and a good peritoneal wash with a large amount of normal saline were routine in these cases, in addition to very strict intravenous and oral antibiotic regimens. Valla *et al*.,[20] recommended the open approach in cases with appendiceal masses. This was not supported in our current study where all of those cases with appendiceal masses were successfully treated laparoscopically. Our findings compared favourably with others in terms of safety and feasibility. Richards *et al*.,[21] reported that laparoscopic appendectomy resulted in fewer complications, a shorter hospital stay and a lower hospital cost than open appendectomy in patients with perforated appendicitis. Chin *et al*.,[22] found laparoscopic appendectomy feasible and safe for complicated appendicitis. The morbidity rate in our series was 15.7%, which was well in the range of these studies.

Tirabassi *et al*.,[23] have reported a considerably high conversion rate (36%) after laparoscopic operation for perforated appendicitis. In our series it was 5%. The conversions occurred in patient with appendicular mass with unhealthy appendicular base and cecal wall gangrene who underwent hemicolectomy.

There are several advantages of the laparoscopic approach in complicated appendicitis. It enables visualisation of the whole abdominal cavity and a thorough peritoneal lavage, which is difficult with a small incision. In open surgery, atypical localisation of the appendix or inaccurate diagnosis may require an extension of the incision as well. The laparoscopic approach also allows patients to become mobile and pain-free much faster, due to less trauma to the muscles and fascia.[24] Another advantage of laparoscopy lies in a 30% lower rate of adhesions, which is a particularly common late complication, especially in children with perforated appendicitis.[25] The benefit of a shorter hospital stay in laparoscopic appendectomy was not observed in our series. This was due to our five-day intravenous antibiotic administration policy. However, the benefit of less post-operative pain and good cosmesis were observed in these patients.

The average length of stay with conservative treatment varied from 10 to 18 days[26,27] with a further eight days required for elective appendicectomy. Our patients had an average stay of six days (6-9 days).
